# MRI Correlates of Depression and Anxiety in Multiple Sclerosis

**DOI:** 10.3390/diseases14070262

**Published:** 2026-07-21

**Authors:** Loredana Sabina Pascu, Simona Dana Mitincu-Caramfil, Andrei Vlad Bradeanu, Ancuța Iacob, Mihaela Lungu, Ileana Marinescu, Eduard Polea Drima

**Affiliations:** 1 Research Centre in the Medical-Pharmaceutical Field, Faculty of Medicine and Pharmacy, “Dunarea de Jos” University of Galati, 800008 Galati, Romania; 2 Faculty of Medicine, University of Medicine and Pharmacy of Craiova, 200349 Craiova, Romania

**Keywords:** multiple sclerosis, depression, anxiety, magnetic resonance imaging, lesion load, gray matter atrophy, functional connectivity, diffusion tensor imaging, neuroimaging, limbic system

## Abstract

Background: Multiple sclerosis (MS) is a neurodegenerative and chronic inflammatory disease, often associated with psychiatric comorbidities, particularly depression and anxiety, which play a major role in disability, cognitive dysfunction, and quality of life. Growing evidence suggests that affective symptoms in MS are not only related to psychosocial burden but also to structural and functional abnormalities in the brain that can be identified using magnetic resonance imaging (MRI). Methods: A systematic database search was performed to identify studies for a structured narrative review. Original articles that examined MRI correlates of depression and anxiety in adult MS patients were included. Structural MRI, diffusion imaging, and functional MRI studies evaluating lesion distribution, cortical and subcortical atrophy, white matter microstructure, and network connectivity were analyzed alongside psychometric instruments used to assess affective symptoms. Results: Depression in MS was consistently associated with fronto-limbic and subcortical abnormalities including cortical thinning, hippocampal and thalamic atrophy, white matter disconnection and altered connectivity within the default mode, salience and executive control networks. Diffusion MRI studies have shown microstructural damage in associative white matter tracts, and functional MRI studies have supported a model of network-level dysfunction. In contrast, anxiety had less robust and reproducible associations with conventional MRI findings, indicating a more complex interaction between inflammatory, neurobiological and psychosocial mechanisms. Conclusions: Multimodal MRI approaches may increase our knowledge of the neurobiological substrates of depression and anxiety in MS and may contribute to more personalized diagnostic and therapeutic strategies.

## 1. Introduction

Multiple sclerosis (MS) is a chronic inflammatory condition that causes inflammation, demyelination, and neurodegeneration of the central nervous system (CNS). Some patients experience remission, during which remyelination and neurogenesis occur in the CNS, leading to a partial recovery. The chronic phase of MS is often associated with permanent neurological dysfunction, primarily caused by axonal degeneration [1,2].

The etiology of multiple sclerosis remains incompletely understood. Current hypotheses emphasize the complex interactions between genetic susceptibility and environmental factors, including autoimmune mechanisms, viral infections, and vitamin D deficiency. Recent evidence has further strengthened the association between Epstein–Barr virus infection and the subsequent development of MS [3].

Histopathologically, MS is characterized by perivascular lymphocytic and macrophage infiltration accompanied by demyelination of nerve fibers within the brain, spinal cord, and cranial nerves (8). Because lesions may involve multiple regions of the central nervous system, their clinical presentations are highly heterogeneous. MRI remains the reference imaging modality for the detection and longitudinal monitoring of MS-related lesions. Typical sites of involvement include the periventricular white matter, callososeptal interface along the inferior surface of the corpus callosum, subcortical U-fibers, brainstem, and spinal cord [4,5].

Although conventional MRI is highly sensitive in detecting lesions associated with multiple sclerosis, it has limited specificity for characterizing lesion severity and underlying pathological processes. It also does not capture the full range of imaging biomarkers required for robust clinical assessment and prognostic stratification of patients. Histopathological–imaging correlation studies have shown that conventional MRI may fail to detect persistent microscopic abnormalities within the normal-appearing white matter, despite the presence of established demyelination, gliosis, axonal injury, or neurodegeneration [6]. More advanced MRI approaches, such as higher-field imaging at 3 T and 7 T [7], as well as quantitative and functional techniques, might allow more accurate characterization of MS-related pathology. These methods may improve the correlation between imaging findings and clinical status, diagnostic specificity for underlying tissue damage, and assessment of processes involved in the accumulation of subsequent disability.

MRI studies of sodium have shown that patients with MS have increased total brain tissue sodium (23Na) concentration. This finding may be linked to cell homeostasis and metabolic integrity in both focal lesions and normal-appearing white and gray matter [7]. Ultra-high-field MRI at 7 T could provide additional information on sodium distribution, including the intracellular sodium component, which has been suggested as a potential biomarker of functional tissue impairment. Transmembrane sodium exchange is a determinant of intracellular sodium concentration and may provide indirect information on cellular metabolism and membrane integrity. In addition, the higher signal-to-noise and contrast-to-noise ratios achieved at 7 T enable improved visualization of anatomical structures and pathological lesions with higher spatial resolution [7,8].

Acute demyelinating lesions may exhibit restricted diffusion on diffusion-weighted imaging. This restricted diffusion may be peripheral or ring-like, corresponding to the actively expanding edge of the lesion. Diffusion restriction is not a uniform feature of acute MS lesions. Following gadolinium administration, active demyelinating plaques may exhibit nodular, ring-like, or incomplete ring enhancement on T1-weighted images [4,9]. Enhancement generally persists for approximately 4–8 weeks and reflects the transient disruption of the blood–brain barrier during active inflammation. Because MS lesions develop at different time points, enhancing and non-enhancing plaques may coexist, providing imaging evidence of dissemination over time [10].

The clinical course and prognosis of MS are heterogeneous and depend on several factors, including the age at onset, presenting symptoms, and disease phenotype. Younger age, optic neuritis or sensory symptoms at onset, and a relapsing–remitting course have traditionally been associated with a more favorable prognosis. Although a substantial proportion of patients remain only mildly or moderately affected, others experience a prolonged disease course, characterized by the progressive accumulation of neurological deficits. Advanced disease may be associated with marked physical disability and cognitive impairment [5,11]. In individuals older than 45 years, periventricular white matter lesions require judicious interpretation because chronic small-vessel ischemic changes may mimic demyelinating plaques, thereby increasing the risk of misdiagnosis [12].

Compared with the 2017 McDonald criteria, the 2024 revision expands dissemination in space by including the optic nerve as a fifth anatomical location, accepts the kappa free-light-chain index as an alternative to CSF oligoclonal bands, and incorporates the central vein sign and paramagnetic rim lesions as supportive imaging biomarkers. The revised framework also reduces the obligatory role of dissemination in time in selected diagnostic pathways and introduces more explicit recommendations for radiologically isolated syndrome, progressive disease, pediatric presentations, and older patients with potential vascular mimics [10].

Normal-appearing white matter (NAWM) describes noninfarcted white matter that appears unaffected on conventional MRI, but may still contain diffuse microscopic abnormalities, including gliosis, axonal injury, demyelination, and metabolic disturbance. Proton MR spectroscopy may provide additional information regarding these changes. Increased myo-inositol levels may indicate glial proliferation, decreased N-acetylaspartate (NAA) may reflect axonal or neuronal dysfunction, while increased glutamate may be linked to excitotoxic injury. During the acute inflammatory phase, increased concentrations of choline, creatine, myo-inositol, and glutamate may be observed in the brain, along with decreased NAA. In chronic diseases, persistent elevation of myo-inositol and reduction in N-acetylaspartate are more commonly reported, consistent with ongoing gliosis and axonal loss [13]. In recent years, a better insight into the pathophysiology of MS has contributed to the development of innovative treatment strategies.

MS impacts more than just movement, sensorium, vision, and cognition. Neuropsychiatric symptom studies have provided new insights into the biology of MS, and provided new therapeutic targets to improve MS patients’ care [14,15]. Anxiety, depression, affective disturbances, hypomanic symptoms and cognitive impairment are the most commonly reported conditions, substantially contributing to the disease burden and reduced quality of life [15,16,17,18,19]. Beyond neurological disability, psychiatric symptoms are increasingly recognized as part of a complex and multidimensional disease burden in patients with MS. Psychiatric manifestations are often associated with broader systemic and functional impairments. Depression, anxiety, alcohol abuse, cognitive decline, and chronic medical comorbidities are all linked through interconnected biopsychosocial and neuroendocrine pathways [20,21,22,23]. Multidisciplinary and patient-centered approaches that integrate psychological, social, and somatic dimensions, especially in vulnerable populations with cognitive impairment, chronic disease burden, or substance-related disorders [24,25].

Psychiatric symptoms are more commonly encountered in relapsing-remitting multiple sclerosis (RRMS) and are now increasingly recognized as an integral component of MS rather than merely a secondary psychological reaction to physical disability [26,27,28]. Emotional instability and introverted personality traits may increase vulnerability to negative affectivity. Psychosocial distress, psychological stress, and persistent negative mood were associated with more frequent relapses than those in patients experiencing a more stable remitting course [29,30,31]. Mood disorders in multiple sclerosis are thought to have multiple causes, including a reactive component and potentially a distinct neuropathological process, which tends to be very common during the early stages of MS [32]. “Pathological hidden changes” such as cognitive impairment, anxiety, depression, and migraines were observed many years prior to the onset of MS and were termed prodromal symptoms [33,34,35,36,37]. Structural MRI may be useful for assessing lesion location and volume, white matter lesion burden and atrophy [38].

This review examines the most relevant MRI findings and ongoing controversies in the current literature to provide a comprehensive understanding of multiple sclerosis.

## 2. Materials and Methods

This study was designed as a structured comprehensive review of the available literature on MRI correlates of depression and anxiety in multiple sclerosis. A literature search was conducted from May 2014 to May 2026 in the PubMed, Web of Science, and Scopus databases, using systematic review methodology, conducted and reported in compliance with the PRISMA 2020 Statement [39]. The following Medical Subject Headings (MeSH) terms and combinations of the following key words were used: for magnetic resonance imaging—“MRI”, “MR”, “structural MRI”, “fMRI”, “DTI”, “structural connectivity” “lesion load”, “gray matter atrophy”, “white matter”, “hippocampus”, “amygdala”, “infratentorial” and for coexisting neuropsychiatric conditions—“depression”, “anxiety”, “neuropsychiatric disorders” along with “multiple sclerosis”, “MS”, “relapsing-remitting MS”, “secondary progressive MS”. To ensure a more comprehensive search, the reference lists of all eligible studies were manually reviewed for additional relevant records. Gray literature sources were not included in the search strategy. The research addressed both qualitative and quantitative studies. The research question was structured according to the PICO framework, reported in Appendix A.

### 2.1. Inclusion/Exclusion Criteria

Primary studies with patients with confirmed multiple sclerosis aged >18 years old, who underwent conventional MRI, functional and/or diffusion MRI scans, and were evaluated using validated psychometric tests for depression and/or anxiety or established diagnostic criteria were included. Cross-sectional, cohort, randomized controlled trials, and quasi-experimental studies on patients with multiple sclerosis with depression and/or anxiety were included.

Criteria for exclusion were non-English articles, review articles, abstracts, editorials, conference papers, meetings, letters, errata, case reports, pediatric studies, and non-human studies.

### 2.2. Data Extraction, Synthesis and Quality Assessment

Two reviewers (P.L.S. and M-C.S-D.) independently screened the titles and abstracts of all identified records according to the inclusion and exclusion criteria to minimize bias in the selection process. Full texts were evaluated if eligibility could not be determined by title/abstract. Duplicate records were removed from both within and across databases. Studies without specific MRI correlations, or studies where patients with MS were only a small subgroup within a heterogeneous cohort, and no separate analysis or extractable data were provided, were excluded. The risk of bias assessment was conducted based on the alignment between each study’s methodological approach and research question, the appropriateness of data collection methods and reports of data analysis, consistency between conclusions and analysis, and the presence of ethical approval. Disagreements were resolved through discussion. The following variables were extracted: study design, sample size, psychometric assessment tools, MRI modality and sequences, assessed brain regions, lesion burden measures (if available), volumetric data, and reported MRI–psychopathological correlations. The evidence was synthesized as a narrative review, due to significant heterogeneity that may prevent pooled data. The review protocol was not prospectively registered in PROSPERO or in another publicly accessible systematic review registry.

## 3. Results

**Figure 1:**
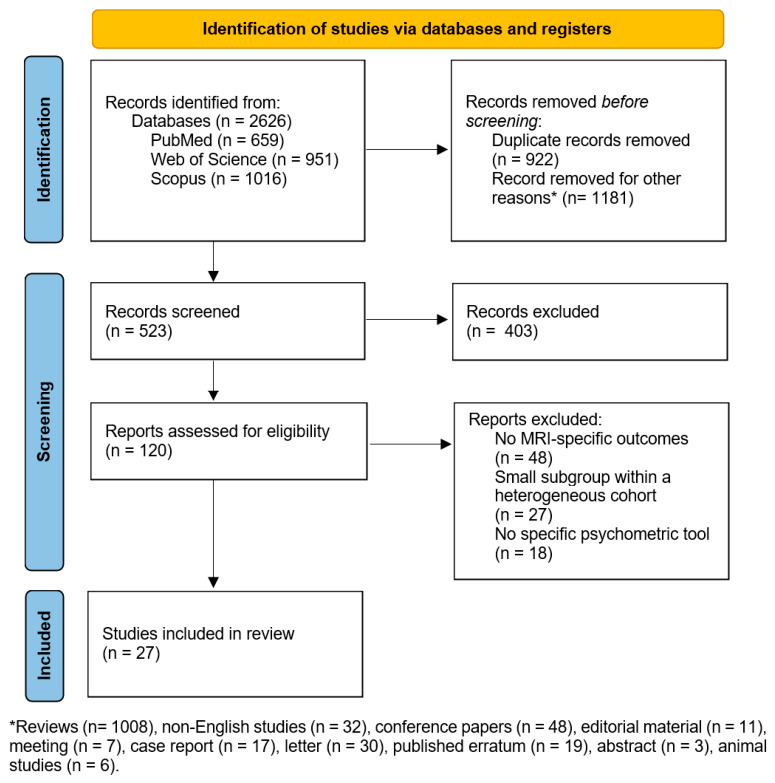
Flow diagram of study selection process. A comprehensive overview of the methodological characteristics, neuropsychiatric assessment methods, MRI techniques, and reported neuroimaging correlates of depression and anxiety in MS in all included studies was provided in Table 1.

The database search retrieved 2626 records. After removing duplicates and excluding non-original study designs, non-peer-reviewed or non-full-text materials, case reports, pediatric populations, and non-human studies, 523 records were assessed by title and abstract. Of the 120 reports, we excluded 18 records with no clearly specified psychometric tool, 27 records where patients with MS were only a small subgroup within a heterogeneous cohort, and 48 records with no specific MRI-derived outcomes. Twenty-seven studies met the inclusion criteria and were included in the review (Figure 1).

**Table 1:** Main MRI correlations between depression and/or anxiety and brain anomalies in PwMS.

Year	Authors	Anxiety Assessment	Depression Assessment	MRI Acquisition	Imaging Variables	Principal Findings
**Depression**						
2024	Baller et al. [40]	N/A	PHQ-2, PHQ-9	3 T 3D T1w 3D FLAIR	WM tracts (fascicles) within a predefined “depression network” Lesion load	Depression was associated with injury to the fascicles inside the white matter depression network.
2022	Kever et al. [41]	N/A	BDI-II	T1w T2w FLAIR	Brain volume: GM, WM Subcortical GM structures (amygdala, basal ganglia, nucleus accumbens, hippocampus, thalamus) Network structure	No association was found between brain measures and depression. Amygdala volume associated with social network size, not mood, suggesting social factors may modulate brain structure independently of depression.
2022	Romanello et al. [42]	N/A	BDI-II	3 T T2w rs-fMRI	Total brain atrophy Lesion load FC: networks—default mode network and attention networks	Depression severity was associated with global connectivity states and DMN-attention network interactions. Dynamic functional measures were more sensitive than static measures. Depression was linked to network-level temporal dysregulation.
2021	Kopchak and Odintsova [43]	N/A	BDI	1.5 T	Brain volume: bifrontal, caudal, ventricular indices, measures subarachnoid spaces (cranio-cortical width, sino-cortical, interhemispheric width), GM (cortical) thickness	Combined lesions of frontal lobe and corpus callosum, frontotemporal region were associated with depression.
2021	Beaudoin et al. [44]	N/A	BDI-II	3 T T1w, T2w, DTI: multi-shell HARDI, tractography	WM microstructure (multiple tracts); Metrics: FA, MD, RD, AFDtot and NuFO, free-water fraction WM bundles volumes, total lesion load, lesion volume in bundles, tractometry	Depression was associated with: - diffusion abnormalities in the right superior longitudinal fasciculus. - lower FA, an higher in RDt and MDt (correlated with severity) Lesion load, WM bundles, free-water fraction and HARDI-derived measures showed no significant clinical association.
2020	Carotenuto et al. [45]	N/A	HDRS	3 T T1w T2w FLAIR rs-fMRI	Whole-brain lesion volume rs-fMRI ROI: serotonergic Noradrenergic cholinergic dopaminergic networks	Depression was not associated with structural modification. Correlated with a reduction in serotonergic and noradrenergic activity and increased cholinergic activity.
2020	Golde et al. [46]	N/A	HADS-D	3 T BOLD, MPRAGE DTI rs-fMRI	Whole and regional (hippocampus, amygdala, fusiform gyrus) brain and GM volumes FC of the fusiform gyrus, occipital cortex, social cognition networks	No association was found with whole-brain volume or structural connectivity measures.
2020	Lazzarotto et al. [47]	N/A	BDI-II	3 T 3D T1w MPRAGE 3D FLAIR 3D DIR	Lesion metrics Supratentorial GM volume Cerebellar GM volume Cerebellar lobular volumes	Depression in MS was associated with selective cerebellar atrophy (posterior cerebellum particularly with vermis Crus I (affective cerebellar region) No association with: white-matter lesion load (WML) gray-matter lesion load (GML) Global supratentorial GM volume
2019	van Geest et al. [48]	N/A	BDI-II HADS-D	1.5 T 3D T1w MPRAGE axial PD/T2w TSE Diffusion EPI rs-fMRI (EPI)	Brain volumes: GM, WM, subcortical Lesion measures: T2LL, tract-specific lesion load DTI metrics: FA, MD	WM volume was reduced in depressed PwMS; Reduced FA in the uncinate fasciculus; Functional connectivity reduced especially between amygdala—frontal cortex
2019	Hassan et al. [49]	N/A	DSM-5 BDI-II	1.5 T T1w, T2w, FLAIR, DTI	FA in Limbic system	Depression was associated with: - decreased FA values in the cingulum, UF, and the fornix; Anterior thalamic radiations had no mean FA differences.
2017	Pravata et al. [50]	N/A	MASDRS	3D T1w	Whole-brain cortical thickness Deep gray-matter volumes Lesion load	Depression was associated with selective cortical thinning in the frontotemporal regions, particularly in the right entorhinal cortex (correlates with depression severity) Depression was associated with focal GM changes. Frontal cortical thinning best predictor of depression.
2017	Rojas et al. [51]	N/A	BDI-II DSM-5	1.5 T T1w, T2w FLAIR DTI	Brain volume Lesion load neocortical GM and T2 lesion volumes FA in corticospinal tract	Depression was associated with increased T2 lesion load and reduced total brain volume. No differences in WM volume and global FA.
2017	Bonavita et al. [52]	N/A	CMDI	3 T rs-fMRI	FC of DMN, SN, ECN ROIs: PCC, ACC, frontal and parietal regions	Depression was associated with functional connectivity alterations, not just structural damage. - Reduced connectivity in PCC (DMN) - Increased connectivity in anterior cingulate and SN regions Overlap between depression and cognitive impairment networks
2016	Yaldizli et al. [53]	N/A	CES-D	1.5 T T1w, PD/T1w	Olfactory bulb volume WM lesion load	Reduced olfactory bulb volume. No association with cognition or lesion load. In progressive MS, olfactory bulb volume was associated with depression severity.
2016	Kallaur et al. [54]	N/A	HADS	T1w, T2w, Gd+		Depression was inversely correlated with gadolinium-enhancing lesions.
2015	Nygaard et al. [55]	N/A	BDI	1.5 T 3D T1w, FLAIR	Cortical volume Lesion load	Depression was linked with reduced cortical surface areas of: - frontal pole, pars orbitalis, and orbital frontal region - rostral, caudal middle frontal, the pre- and post-central regions - left temporal regions: middle temporal, fusiform and parahippocampal regions. Reduced volumes of: - orbital frontal and pars orbitalis, the superior frontal - rostral and caudal middle frontal, pre- and post-central regions - right supramarginal and superior temporal regions - left fusiform and inferior temporal region. The association with depression was independent of lesion load.
2015	Nigro et al. [56]	N/A	DSM-5 BDI-II	T1w FLAIR DTI	GM, WM volumes Lesion load Tractography WM connectivity	No structural modifications correlated with depression. Depression correlated with increased shortest-path length between: - right hippocampus—right amygdala; - dorsomedial and ventrolateral PFC—the occipitofrontal cortex.
2014	Štecková et al. [57]	N/A	BDI-II	1.5 T T1w 3D T1w MPRAGE	Thalamus volume Global brain volume	Depression scores were associated with reduced thalamic volume 5 years after diagnosis and increased volume 10 years after diagnosis. Thalamic atrophy was more related to cognitive impairment than to depression.
**Depression and anxiety**						
2025	Baller et al. [58]	PHQ-2 PHQ-9	PHQ-2 PHQ-9	3 T 3D T1w 3D FLAIR	Uncinate fasciculus Amygdala orbitofrontal cortex Lesion volume	Anxiety presence and severity correlated with lesion load in the UF. Depression was not associated with lesion load in UF or fornix damage. Total lesion load was associated with general psychopathology but does not specifically correlate with anxiety, suggesting that both diffuse brain injury and targeted disruption of fronto-limbic circuits play roles in psychiatric manifestations in MS.
2025	Giofre et al. [59]	SPECTRA	SPECTRA	3 T 3D T1w MPRAGE 3D FLAIR AX T2w DWI	Lesion metrics Infratentorial lesion load topography Brain volume	Depressive symptoms were significantly associated with the presence of lesion load within: - raphe nuclei (serotonergic system) - locus coeruleus (noradrenergic system) - medial midbrain - medial pons No association between depression and cerebellar lobule VIIA or brain volume. No significant associations between anxiety and lesion load and brain volumes. Lesion load in locus coeruleus did not reach statistical significance.
2023	Hillyer et al. [60]	HADS-A	HADS-D	7 T T1w MPRAGE T2 FLAIR 3D multi-echo GRE MPR2-RAGE DIR-SPACE	Limbic system lesion localization and metrics	Anxiety was associated with the presence of lesions in the limbic system, but not with lesion load or volume. Depression was not associated with limbic lesions.
2023	Riemer et al. [61]	HADS-A	HADS-D	3 T 3DT1 MPRAGE, T2, FLAIR; Diffusion-based FWF analysis	Brain volumes (subcortical, WM, GM) Subcortical structures FWF	Depression was associated with higher FWF in the amygdala-hippocampus, thalamus, corpus callosum, cerebellar vermis, and precuneus. Subcortical microstructural abnormalities, especially in the thalamus and limbic system, preceded depression (2 years). Anxiety was weakly correlated with the structural findings.
2018	Palotai et al. [62]	STAI	BDI	3 T 3D T1w 3D FLAIR	Lesion mapping and connectome-based tract analysis	Anxiety was associated with WMLL in the column and body of the fornix.
**Anxiety**						
2025	Meyer-Arndt et al. [63]	STAI-T	N/A	3 T T1w FLAIR fMRI (T2*w EPI), Spin-echoEPI Multi-shell DWI	Whole-brain MVPA (SVR) GM volume fraction Graph-based connectome Hippocampus, anterior insula, inferior temporal gyrus	Anxiety was linked to functional alterations in fear-processing networks (fMRI) and reduced structural connectivity (DWI), particularly hippocampus and anterior insula.
2025	Nyári et al. [64]	STAIS-5 STAIT-5	N/A	3 T T1w FLAIR	Lesions count T1 “black holes”	No significant association was found between MRI lesion measures (including infratentorial lesions) and anxiety (state or trait). Anxiety was highly related to depression, fatigue and secondary progressive disease.
2022	Ellwardt et al. [65]	HADS-A	N/A	3 T 3D T1w 3D FLAIR rs-fMRI	Brain volume Lesion load Structural findings left dorsal prefrontal cortex Atrophy network mapping; Prefrontal cortex Amygdala Hippocampus	Anxiety was highly correlated with cortical thinning in the left dorsal PFC. Volumes of PFC, amygdala and hippocampus predicted anxiety severity. Anxiety was correlated with: - reduced connectivity between PFC and amygdala. - compensatory increase between hippocampus and PFC. - rs-fMRI: Increased PFC excitability at rest and reduced response under threat.
2019	Chalah et al. [66]	HADS-A	N/A	3D T1w VBM-like structural analysis	GM, WM volumes Regional volumes normalized to total intracranial volume	Anxiety scores was not associated with regional GM/WM volumes. Large caudate nuclei and reduced left parietal cortex volume in patients with anxiety and fatigue—linked to fatigue, not to anxiety per se.

Abbreviations: ACC, anterior cingulate cortex; AFDtot, total apparent fiber density; BDI, Beck Depression Inventory; BDI-II, Beck Depression Inventory-II; BOLD, blood oxygen level-dependent; CES-D, Center for Epidemiologic Studies Depression Scale; CMDI, Chicago Multiscale Depression Inventory; DIR, double inversion recovery; DMN, default mode network; DSM-5, Diagnostic and Statistical Manual of Mental Disorders, Fifth Edition; DTI, diffusion tensor imaging; DWI, diffusion-weighted imaging; ECN, executive control network; EPI, echo-planar imaging; FA, fractional anisotropy; FC, functional connectivity; FLAIR, fluid-attenuated inversion recovery; FWF, free-water fraction; Gd+, gadolinium-enhancing; GM, gray matter; GRE, gradient-recalled echo; HADS, Hospital Anxiety and Depression Scale; HADS-A, HADS anxiety subscale; HADS-D, HADS depression subscale; HARDI, high-angular-resolution diffusion imaging; HDRS, Hamilton Depression Rating Scale; MD, mean diffusivity; MDI-FS, Major Depression Inventory-Fast Screen; MPRAGE, magnetization-prepared rapid acquisition gradient echo; MPR2RAGE, magnetization-prepared 2 rapid acquisition gradient echoes; MS, multiple sclerosis; MVPA, multivariate pattern analysis; N/A, not assessed; NuFO, number of fiber orientations; OFC, orbitofrontal cortex; PCC, posterior cingulate cortex; PD, proton density; PFC, prefrontal cortex; PHQ-2, Patient Health Questionnaire-2; PHQ-9, Patient Health Questionnaire-9; PwMS, people with multiple sclerosis; RD, radial diffusivity; ROI, region of interest; rs-fMRI, resting-state functional MRI; SN, salience network; STAI, State-Trait Anxiety Inventory; STAI-T, State-Trait Anxiety Inventory-Trait; STAIS-5, five-item state anxiety scale; STAIT-5, five-item trait anxiety scale; SVR, support vector regression; T1w, T1-weighted; T2w, T2-weighted; T2LL, T2 lesion load; TSE, turbo spin echo; UF, uncinate fasciculus; VBM, voxel-based morphometry; WM, white matter; WML, white-matter lesion; WMLL, white-matter lesion load.

### 3.1. Depression

#### 3.1.1. Epidemiology and Clinical Impact

Among all psychiatric comorbidities in MS, depression is the most prevalent and extensively studied condition. It was three to five times more frequent than in the general population [67], underscoring the disproportionate mental health burden experienced by individuals with MS. It ranged from 18.5% [68] to 58.9% [69] among PwMS, reflecting the heterogeneity in research populations, diagnostic methods, and assessment tools. According to epidemiological findings, the rates of depression in PwMS vary significantly across Europe. A study conducted in Spain found an overall prevalence of 44%, yet there were marked differences by MS subtype—with a striking 88% in relapsing-remitting MS (RRMS), versus 12.2% in secondary progressive MS (SPMS) [70]. In England, a large cohort study reported that 21% of PwMS exhibited depression at baseline, in contrast to 9% of healthy controls [71]. Another multicenter German study identified at least minimal depressive symptoms in 32.8% of patients, with 16.1% with mild symptoms and 15.9% with severe depression [72].

Depressive symptoms were closely associated with inflammatory disease activity. Depression was more frequently observed in the relapsing phases of increased neuroinflammation, particularly during clinical relapses, and was more common in the early years following disease onset. This temporal association supported the hypothesis that inflammatory mechanisms, rather than psychosocial factors alone, contribute to a significant association in the development of depressive symptoms. With aging, depressive symptoms tend to decrease, as neurodegenerative processes becoming more prevalent, suggesting a change in the biological factors that affect mood regulation [73]. Recent research on metabolic and oxidative stress pathways provided a useful biological framework for understanding affective symptoms as multidimensional and not purely reactive manifestations of complex chronic disorders, highlighting the broader role of inflammation, endocrine imbalance, and oxidative injury [74,75].

Depression is considered a debilitating comorbidity and associated with decreased quality of life, disability progression, cognitive impairment, poor treatment adherence, and increased fatigue [76], even though fatigue is considered both a symptom and a complication of depression [73]. Emerging data also suggest that chronic stress, depression and alcohol-related behavioral disturbances influence complex neurobiological and inflammatory changes, including hypothalamic–pituitary–adrenal axis dysregulation, impaired healing, metabolic dysfunction and reduced functional outcomes [20,21,22].

Furthermore, it remains under-recognized and undertreated, with approximately 23–30% of cases going undiagnosed and 20–36% of diagnosed patients receiving insufficient treatment [77].

#### 3.1.2. Imaging Findings

**Structural MRI**

Depressive symptoms were highly correlated with psychometric tests, lesion load on T2-weighted sequences, and globally reduced volume [48,51], compared to non-depressed patients. Depression was associated with structural GM abnormalities rather than WM lesion load [55]. It was linked to reduced cortical surface area and volume, especially in the frontal pole, pars orbitalis orbitofrontal cortex, and rostral and caudal segments of the middle frontal gyrus, and precentral and postcentral regions. Additional involvement was observed in the left temporal regions, such as the middle temporal, fusiform, and parahippocampal regions [55]. Reduced volumes were observed predominantly in the orbitofrontal cortex and pars orbitalis, along with the superior frontal cortex, rostral and caudal middle frontal gyri, and precentral and postcentral areas, right supramarginal and superior temporal regions, and left fusiform and inferior temporal cortices [50,55], highlighting frontal cortical thinning as the best predictor. Additionally, depression severity has been linked to reduced olfactory bulb volume in patients with progressive MS [53]. Cortical thickness was not significantly correlated with depression severity, suggesting that depression was more related to alterations in surface area and volume than to diffuse cortical thinning, and it also remained partly independent of WM lesion load [41,46,53,55]. Other recent studies highlighted that reduced gray matter volume in the right prefrontal cortex [78], and in the right dorsolateral and inferior frontal cortices (using voxel-based morphometry) [79] predicted subsequent worsening, or lack of improvement, in depressive symptoms.

A longitudinal study [78] demonstrated that increased volume loss of the left thalamus and right internal globus pallidus, and decreased GM volume in the right middle cingulate cortex and middle frontal gyrus were predictive of subsequent worsening of depressive and anhedonic symptomatology. Thalamic atrophy in both patient groups, compared to healthy controls, indicated this change is more correlated to MS-related neurodegeneration than a depression-specific process.

The limbic motivational basal ganglia circuit contains interconnected structures, including the mediodorsal thalamus and internal globus pallidus [80]. The globus pallidus is a key node in the dopaminergic reward-processing circuit [81], which can serve as a conceptual reference to contextually understand the association between its progressive atrophy and the longitudinal course of depressive symptoms. Structural degeneration of the globus pallidus compromises its ability to encode reward-related information in the brain. This leads to core depressive features such as apathy and anhedonia. From a clinical perspective, it was noteworthy that anhedonia linked to pallidal dysfunction may be, at least partially, reversible through dopaminergic modulation [78].

Progressive damage to subcortical motivational systems represented a risk factor for the development of depression in people with MS, whereas preserved prefrontal function may act as a protective or resilience factor [78].

Depression was correlated with infratentorial lesions [59,82] and was strongly correlated with damage to serotonergic and noradrenergic brainstem nuclei, the raphe nuclei, and the locus coeruleus, overlapping with neuropathological findings in Parkinson’s disease [83,84]. Depression was correlated with medial midbrain and medial pons lesions, but no association was observed with cerebellar lobule VIIA [59].

The association of depressive symptomatology in patients with RRMS to selective atrophy of posterior cerebellar structures, especially vermis Crus I, rather than white or gray matter lesion burden, supported a network-based model of affective dysfunction involving cerebello-limbic circuits was supported by the fact that was linked to [47].

A retrospective study by Hillyer et al. [60] found no significant association between depressive symptomatology and the presence, number, or volume of lesions within limbic system structures on 7T MRI. These results were consistent with previous limbic-focused research [56,85]. Notably, Berg et al. [85] reported that people with MS (PwMS) and depression exhibited an increased lesion burden in cortical projection regions linked to the limbic system (the temporal, frontal, and parietal lobes), while no corresponding increase was observed within the limbic system.

White matter lesions had similar patterns in patients with vascular depression, supporting the hypothesis of common underlying pathophysiological mechanisms contributing to secondary forms of depressive disorders [86].

**Diffusion tensor imaging**

Diffusion tensor imaging (DTI) is a new, non-invasive MRI method that allows assessment of the brain microstructure. Fractional anisotropy (FA) measures the extent of water movement in different directions within tissue. Lower FA values indicate white matter abnormalities caused by axonal damage. DTI provides enhanced specificity in the assessment of multiple sclerosis (MS) compared to conventional MRI because of the heterogeneous characteristics of its underlying pathology [87]. It enabled the characterization of tissue damage not only in overt white matter lesions, but also in normal-appearing white matter (NAWM) and gray matter (NAGM), as well as in the optic nerves and spinal cord. Changes such as lower FA and increased mean diffusivity may occur before visible lesions on conventional MRI [49,88].

Diffusion imaging studies demonstrated reduced FA within the NAWM in PwMS and depression, and increased mean diffusivity in the NAGM, particularly in the temporal lobes and inferior frontal regions. These modifications were associated with cortical atrophy in the bilateral frontal areas [49,89,90,91].

Baller et al. found a link between depression and MRI data, using lesion network mapping. Associations with occurrence and severity of depression were stronger for the lesion load within specific depression-associated white matter tract segments, compared to total lesion volume [40].

In PwMS, the development of depression was associated with disruption of the limbic-motor interface, and broader network disconnection, as demonstrated by alterations in the structural connectivity between the right hippocampus and amygdala, and frontal regions [56,91]. Some studies [48,56] highlighted alterations in structural network organization, specifically increased path length between the amygdala and hippocampus and the prefrontal cortex, associated with structural changes with decreased amygdalar volumes, suggesting impaired efficiency of information transfer across these regions. Previous studies showed that conscientiousness and WM pathology in MS were connected, with specific fronto-parietal networks being disrupted. These networks included structural connections linking the frontal, fronto-cingulate, frontoparietal, and basal ganglia gray matter (GM) regions [91,92]. Those regions were associated with executive control and the formation of associations between actions and rewards [93]. Given the well-established association between conscientiousness and depression, it was hypothesized that WM tract disruption within conscientiousness-associated frontoparietal networks (CFPN) may serve as a predictor of incident, clinically diagnosed depression over a 5-year period in PwMS [92]. In addition, van Geest et al. showed that depressed MS patients had reduced FA in the uncinate fasciculus [48], consistent with previous studies [49], where a significant reduction in the FA of the cingulum and fornix was additionally noted.

**Functional MRI**

Functional MRI (fMRI) in patients with MS and depression demonstrated impaired functional connectivity (FC) within reward circuits, fronto-limbic, and fronto-striatal networks, frequently indicating diminished connectivity between the prefrontal cortex and amygdala. These changes were linked to the severity of depression and brain inflammation, showing that depression related to MS was caused by brain damage (lesions and atrophy) rather than just a mental response to the disease, which was correlated with widespread network-level alterations [91,94,95,96].

Functional connectivity alterations are associated with reorganization of the brain network in response to MS-related damages, with increased compensatory connectivity [45,91].

The link between depressive symptoms and atypical connectivity was evident, particularly in the serotonergic and noradrenergic systems. The connectivity pattern involved the brainstem nuclei, cerebellum, and their projections to the limbic and frontal regions, pointing to a neurotransmitter-driven network mechanism underlying depression [45].

Resting-state fMRI analyses showed that changes in connectivity extend beyond the usual fronto-limbic circuits. They also include areas such as the cerebellum and hypothalamus, which are involved in regulating emotions and the autonomic nervous system, adding to the neurobiological understanding of depression in patients with MS [45,91,97].

Van Geest et al. showed that depressed MS patients had reduced functional connectivity between the amygdala and frontal regions. Disease duration, fractional anisotropy of the left uncinate fasciculus, and functional connectivity of the right amygdala accounted for 48% of the variance in depression severity [48].

### 3.2. Anxiety

#### 3.2.1. Epidemiology and Clinical Impact

Anxiety is another prevalent psychiatric symptom in PwMS, with a prevalence varying from 19.3% [98] to 58% [99,100]. Anxiety was most frequently observed at the peri-diagnostic time [101], as well as during phases of clinical relapse, associated with neurological disability, uncertainty related to the course of the disease, treatment options, and quality of life disturbances [102,103]. At this stage, there may be increased emotional discomfort associated with adjustment to a newly diagnosed chronic neurological disorder. Anxiety negatively impacts the patient’s quality of life and can intensify pain and fatigue [104]. Anxiety was approached as a single psychopathological symptom, in most of the previous studies [105,106,107], but it should had been distinguished [64,80,84,88] into state anxiety, a temporary context-dependent condition, and trait anxiety, a more stable tendency to perceive and respond to environmental burden with persistent worry and fear [101,108]. The evolution of both anxiety and depression was influenced by disease-related biological mechanisms, and individual lifestyle and personality factors [109]. Recent studies suggested that personality traits played a meaningful role: higher levels of neuroticism were linked to greater vulnerability to developing depression and anxiety, whereas traits such as extraversion and openness may confer a degree of psychological resilience in individuals with MS [110].

State anxiety correlated with cerebrospinal fluid levels of interleukin-2, and increased severity of state anxiety and depressive symptoms correlated with MS activity. This association was present even in cases of subclinical inflammation on MRI, and psychiatric symptoms were reduced following the resolution of the inflammation [111]. In contrast, trait anxiety did not seem to be affected by inflammatory status, further supporting a specific relationship between neuroinflammation and transient affective disturbances in patients with MS. Emotional symptoms with sudden onset or worsening may be potential indicators of underlying disease activity, emphasizing the importance of careful evaluation in such contexts [101].

#### 3.2.2. Imaging Findings

**Structural MRI**

Anxiety in PwMS showed no consistent relationship with lesion load, specific lesion distribution (infratentorial lesions) or regional atrophy on conventional MRI, unlike depression [58,60,61,64,66]. Anxiety was strongly related to depression, fatigue, and secondary progressive disease [61,112]. Studies that focused on limbic and brainstem structures did not yield robust and reproducible findings. While subtle alterations in limbic connectivity or cognitive performance were reported, these did not seem sufficient to delineate a clear neuroanatomical substrate. Even if a higher incidence of the lesion load in the locus coeruleus (noradrenergic system) in patients with anxiety than in those without was described, it did not reach statistical significance [59]. No correlations were found between lesion load in raphe nuclei (serotonergic system) and cerebellar or brainstem lesion topography [59]. Instead, anxiety in MS was likely influenced by a combination of psychological, cognitive, and contextual factors, including disease uncertainty and stress-related responses. Collectively, these findings supported a model in which depression in MS was more closely linked to structural and network-level brain damage, whereas anxiety appeared to reflect a more complex and less structurally determined interplay between neurobiological and psychosocial mechanisms [63,113].

Anxiety levels were associated with higher normalized brain volumes, in contrast to the typically reduced brain volumes in PwMS [59]. This was explained by the pathophysiology of anxiety, which is less driven by neurodegeneration and more influenced by psychological and behavioral factors than depression.

Generalized anxiety disorder was also studied in late-life patients, and it was correlated with reduced cortical thickness in orbito-frontal cortex, anterior cingulate cortex and inferior frontal regions, areas involved in emotional regulation and threat appraisal; however, after adjustments, only the left rostral anterior cingulate cortex was statistically significantly correlated. Additionally, the brain volumes in the right inferior frontal gyrus, pars triangularis and pars opercularis correlated moderately, and no association was seen between WM hyperintensity burden, WM integrity of diffusivity and anxiety [113]. In a retrospective study by Hillyer et al. [60], performed using 7T MRI, a significant relationship between the presence and volume of lesions in the limbic system and anxiety was observed.

PwMS with fatigue and severe anxiety exhibit increased caudate volume and reduced cortical thickness in the left parietal cortex compared to those without fatigue [66].

**Diffusion tensor imaging**

The correlation between anxiety and lesion topography in limbic white matter circuits, particularly the septo-fornical pathway, column, and body of the fornix [62], was evidenced using lesion mapping. That pointed to a substrate at the level of the limbic circuit that is distinct from global white matter lesion burden.

Anxiety was correlated with reduced structural connectivity, predominantly in the hippocampal and anterior insula regions, driven by fear overgeneralization [63]. FA was lower in the left cingulum and right uncinate fasciculus and negatively correlated with anxiety severity, whereas FA values were not correlated with anxiety in the general population [113], suggesting that anxiety in PwMS has an underlying WM tract pathophysiology.

**Functional MRI**

A combined longitudinal assessment of cortical atrophy on conventional MRI with normative rs-fMRI connectivity mapping to identify an anxiety-related prefrontal-limbic network suggested that anxiety symptoms were driven by network-level dysfunction rather than isolated structural damage [65]. Task-based fMRI highlighted functional alterations in fear-processing networks modulated by hippocampal-insular circuits, with an underlying mechanism based on generalized fear [63]. Complementary structural studies associated greater uncinate fasciculus (UF) lesion burden with more severe anxiety, independently of total lesion volume, whereas fornix injury was not significantly related to anxiety severity [58,62,114].

**Figure 2:**
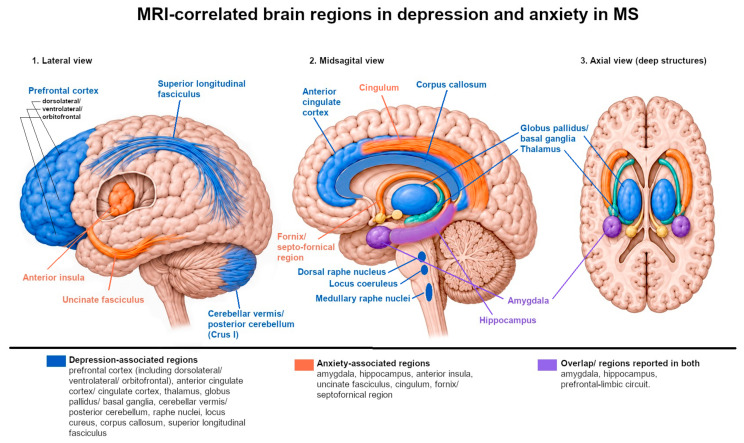
Schematic comparison of the principal brain regions and neural pathways associated with depression and anxiety in multiple sclerosis.

Figure 2 provides a schematic comparison of the main brain regions and neural pathways reported to be associated with depression and anxiety in patients with multiple sclerosis, highlighting both condition-specific and overlapping MRI correlates.

## 4. Discussion

The most consistent finding across the reviewed literature was that affective symptoms in MS were more closely related to the disruption of distributed neural circuits than to the total lesion burden [40,45,48,52,56,58,62,63,65]. This pattern was particularly evident in the cases of depression. Overall, structural MRI studies implicated the frontal, temporal, limbic, subcortical, and cerebellar regions associated with emotional regulation, reward processing, motivation, and cognitive control. The most common structural alterations reported were decreased cortical surface area or volume in the prefrontal, orbitofrontal, middle frontal, entorhinal, fusiform, and parahippocampal regions. Changes in the thalamus, globus pallidus, hippocampus and posterior cerebellum were also reported. These findings supported the view that depression in MS was a disorder linked to fronto-limbic and motivational networks, rather than a simple consequence of diffuse lesion accumulation [40,45,47,48,49,50,51,52,53,55,56,59,61,78,79,80,81,96,97,115,116]. This network pattern was particularly conspicuous in studies on RRMS and early MS. Studies confined to RRMS or early relapse-onset groups found abnormalities in dynamic functional connectivity, associative white matter tracts, cortical morphology, and limbic function. That suggested that circuit disruptions might cause symptoms before widespread neurodegeneration becomes the dominant pathology [100,101,102,103,104]. In early or relapse-onset cohorts, selective posterior cerebellar atrophy and frontotemporal cortical changes were often independent of the total lesion burden [47,55].

Diffusion MRI studies showed a fairly coherent disruption in the microstructure of WM tracts, particularly in the associative and limbic WM tracts [40,44,48,49,56,61,116]. Decreased fractional anisotropy and increased diffusivity were reported in the uncinate fasciculus, cingulum, fornix, and superior longitudinal fasciculus [44,48,49]. These WM tracts link the prefrontal cortex with the limbic and associative areas in accordance with abnormalities in mood regulation, reward processing, and executive control functions [48,49,56,91,92,93,116]. Lesions in defined depression-related pathways were associated with the presence or severity of depressive symptoms, but rather total lesion burden [40,48,56,116].

The functional MRI findings matched the network-based model. Altered connectivity was reported between the amygdala, hippocampus, prefrontal cortex, anterior cingulate cortex, and brainstem neuromodulatory systems [45,48,96,97,115]. There were also noted abnormalities in the default mode, salience, executive control, reward, serotonergic, noradrenergic, and cerebellar networks [42,45,52,94,95,96,97].

These studies enhanced the importance of fronto-limbic and fear-processing pathways in anxiety, although the evidence was not as comprehensive as for depression [58,60,62,63,65]. Abnormalities were reported in the UF, fornix, cingulum, amygdala, hippocampus, anterior insula, ACC, and DLPFC. The functional neuroimaging data indicated altered connectivity between the PFC and amygdala, as well as the hippocampus and PFC in threat appraisal and fear generalization networks [63,65]. Overall, the findings supported regional and connectivity abnormalities as potential MRI correlates of anxiety over global atrophy and total lesion burden.

The major point of contention was the link between affective symptoms and conventional MRI parameters. Depression was associated with various brain changes such as increased T2 lesion load, reduced overall brain volume, regional cortical atrophy, and lower WM volume [48,50,51,55]. In contrast, several studies reported no significant relationship between depression and global lesion load, total GM or WM volume, or conventional structural connectivity measures [41,46,47,56]. Those discrepancies indicated that global MRI measures may not be sensitive enough to detect focal abnormalities in mood-regulating networks. Although some studies linked thalamic atrophy to depression, others indicated that thalamic atrophy was more of a marker of broader MS-related neurodegeneration or cognitive impairment, rather than a depression-specific substrate [55,57,61,78]. Likewise, several studies showed hippocampal and amygdalar abnormalities, using methods assessing network topology and diffusion, while conventional volumetric studies failed to demonstrate mood-specific changes [41,46,48,56,61].

The cerebellar findings were inconsistent too. Depression was linked to selective posterior cerebellar atrophy, especially in affective cerebellar regions; however, other studies found no relationship between depressive symptoms and cerebellar lobular volume or infratentorial lesion load [47,59]. The brainstem findings followed a similar pattern. Lesions involving the raphe nuclei, locus coeruleus, medial midbrain, and pons were associated with depression [45,59,82,83,84]. The significance of some individual nuclei was not always statistically robust [59].

RRMS and early-MS studies identified focal cortical, tract-specific, or functional network abnormalities more often, while correlations with global brain atrophy and lesion burden, which emerged in mixed cohorts including patients with progressive disease, were less consistent [41,42,43,44,45,46,47,48,53,55,56,61,100,101,102,103,104]. In progressive MS, depression and MRI measures were strongly correlated with cumulative neurodegeneration and regional atrophy, as reduced olfactory bulb volume was associated with depression severity only in a subgroup of patients with progressive MS [53]. Likewise, longitudinal findings involving the thalamus, globus pallidus, and other subcortical reward-motivational centers suggested that later-stage depression was more related to degeneration of reward and motivational circuits, rather than to active inflammatory lesion formation [57,61,78,80,81].

The literature on anxiety was even less consistent. Several studies failed to identify an association between anxiety and lesion count, total lesion load, infratentorial lesions, regional gray- or white-matter volume, or T1 hypointense lesions [59,61,64,66]. Other studies reported significant associations with limbic lesions, fornix involvement, uncinate fasciculus lesion burden, cortical thinning in the dorsal prefrontal cortex, and reduced structural connectivity involving the hippocampus and anterior insula [58,60,62,63]. Volumetric findings were inconsistent as well. While some reports describe reduced cortical thickness or regional volume, others found higher normalized brain volume in patients with anxiety, suggesting that anxiety might not follow a simple neurodegenerative pattern [63,65]. The relationship between anxiety and progressive disease appeared to be predominantly clinical rather than radiological.

Anxiety was related to secondary progressive MS, fatigue, and depression, but conventional MRI lesion measures were not independently associated with these variables, [64,91]. Similarly, comparisons between the progressive MS and the relapsing–remitting MS cohorts demonstrated that anxiety burden may vary by phenotype, but those differences were heavily confounded by disability, fatigue and psychosocial factors [86]. Therefore, a higher prevalence or severity of anxiety in progressive MS cannot be used as evidence of a distinctive MRI pattern in progressive MS [64,86,91].

Another debated issue was the relationship between damage to the fornix and anxiety. Lesion mapping studies implicated the column and body of the fornix in anxiety, whereas other studies did not find a correlation between fornix injury and anxiety severity [58,62]. Similarly, a high-field 7 T study found limbic lesions associated with anxiety, whereas conventional field-strength studies generally did not find a consistent relationship between limbic lesion burden and anxiety [60,64,66].

The divergent findings could be attributed to several methodological factors. The first was the heterogeneity of the psychiatric assessment. Some studies assessed depression using instruments such as the BDI, BDI-II, HADS-D, HDRS, CES-D, CMDI, PHQ-2, and PHQ-9. Other studies used DSM-based diagnoses. These tools differ in their sensitivity, threshold for diagnosis, and contribution of somatic symptoms (e.g., fatigue, sleep disturbance and impaired concentration) to the total score [67,68,69,70,71,72,73]. Symptoms in MS might represent neurologic disease rather than depression and inflated associations in some cohorts [67,73,76].

Anxiety assessment was even more heterogeneous in nature. Some studies used HADS-A, others the STAI, or abbreviated state and trait inventories, and some did not distinguish between transient state anxiety and stable trait anxiety [58,60,62,63,64,65,66,80,88]. State anxiety was subject to change depending on inflammatory activity, relapse, diagnostic uncertainty, and recent diagnosis. In contrast, trait anxiety was more strongly driven by personality and long-term psychosocial vulnerability [45,80,84,88].

The second largest source of variability was associated with the type of imaging method used. The studies varied in field strengths (1.5 T to 7 T), spatial resolution, lesion detection sensitivity, sequence selection, and susceptibility to artifacts. Conventional T1- weighted and T2-weighted imaging, FLAIR, volumetric measurements, voxel-based morphometry, diffusion tensor imaging, high-angular-resolution diffusion imaging, tractography, resting-state functional MRI, task-based functional MRI, lesion-network mapping and free-water imaging each provided different information on aspects of disease pathology. A negative study on conventional MRI could be considered compatible with a positive result obtained either by diffusion or functional MRI, since these techniques assessed different biological processes.

The analytical strategies used varied widely among the studies. Some studies focused on global brain volume or overall lesion burden, while others focused on specific regions, tract-specific lesions, cortical thickness, connectivity networks, or even dynamic functional connectivity. ROI-based analysis techniques identified focal abnormalities that were diluted when measured using whole-brain techniques. Small sample sizes increased the risk of false-negative and unstable positive findings. Differences in correction for multiple comparisons, intracranial volume, age, disease duration, disability, treatment status, fatigue, cognition and vascular comorbidities further limited comparability.

The clinical heterogeneity was also significant. The included cohorts comprised RRMS, SPMS, progressive, early-MS, and mixed-phenotype populations with broad age, disease duration, disability, inflammatory activity, and extent of neurodegenerative damage. Most imaging data were obtained from cohorts of RRMS [42,43,44,55,61], early or relapse-onset MS [47,51,65], or mixed cohorts with predominantly relapse-onset disease. Progressive phenotypes were generally included within mixed cohorts or examined in subgroup analyses, as in the studies by Yaldizli et al. [53] and Nyári et al. [64] rather than being investigated in independent SPMS-only or PPMS-only imaging cohorts. Therefore, reported focal network abnormalities in younger RRMS populations were obscured in mixed or progressive cohorts by diffuse atrophy and accumulated structural damage [53,57,61,78].

Depression and anxiety often co-occurred, and both were linked to fatigue, cognitive impairment, pain, relapse activity, medication exposure, and psychosocial stress [45,64,68,80,85,86,87,88,89,91,98,99]. Many studies failed to account for these factors properly, which led to the confounding of general disease-related or behavioral effects with a particular psychiatric symptom [45,64,66,80,85,86,87,88,89,91]. In progressive MS, the accumulation, fatigue, decreased autonomy and chronic symptom burden had a greater impact on affective symptoms than on focal MRI abnormalities [64,86,91].

## 5. Limitations

This review had several limitations. The included studies had considerable heterogeneity in terms of imaging techniques, acquisition parameters, analytical approaches, study population, and adjustment for relevant clinical confounders, as well as a lack of a standardized reporting system. The incidence of RRMS and early MS was higher compared to progressive forms, limiting the generalizability of the results to SPMS and PPMS. Such methodological differences prevented the characterization of MRI-specific markers for depression or anxiety in MS.

## 6. Conclusions

Depression and anxiety are frequently reported in PwMS. Conventional structural MRI findings revealed a limited association with GM atrophy in patients with depression and partial independence from lesion load, while anxiety demonstrated weaker structural correlations. Advanced MRI studies provided stronger evidence of disrupted connectivity within the limbic, prefrontal and neuromodulation circuits. These findings enhance the knowledge about the neurobiological underpinnings of affective symptoms but do not currently support the use of MRI as an independent biomarker for depression or anxiety in MS. The relationship between MS and neuropsychological and neuropsychiatric symptoms remained unclear and multifactorial, including structural and functional brain abnormalities, inflammation, and psychosocial factors.

Future studies using larger cohorts with standardized protocols for conventional and advanced MRI may reveal the link between brain alterations and neuropsychiatric symptoms. Currently, MRI findings are considered only exploratory markers without validated clinical biomarkers for diagnosis, prognosis, or treatment selection.

## Figures and Tables

**Figure A1:**
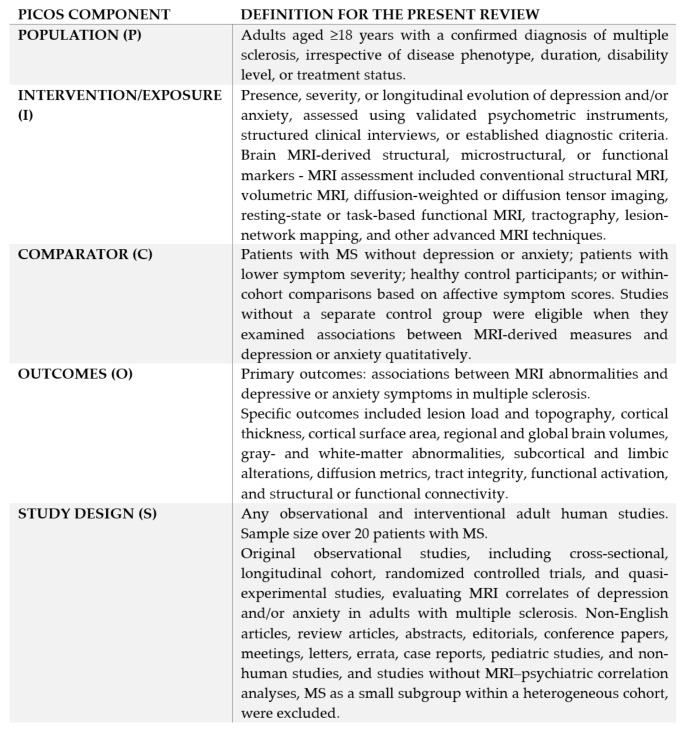
PICOS criteria to define the research questions.

**Figure A2:**
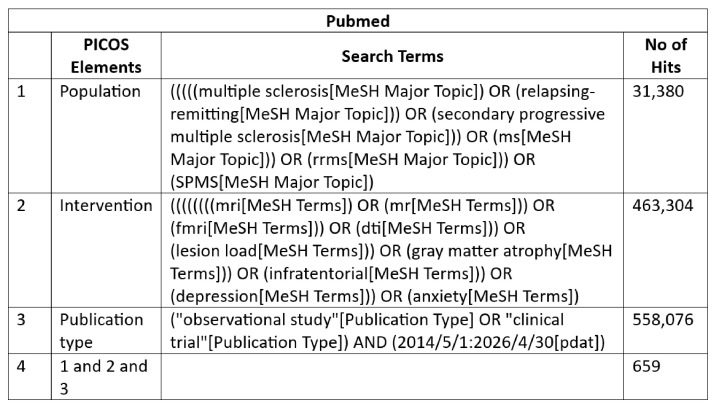
Search strategy and number of hits in PubMed.

**Figure A3:**
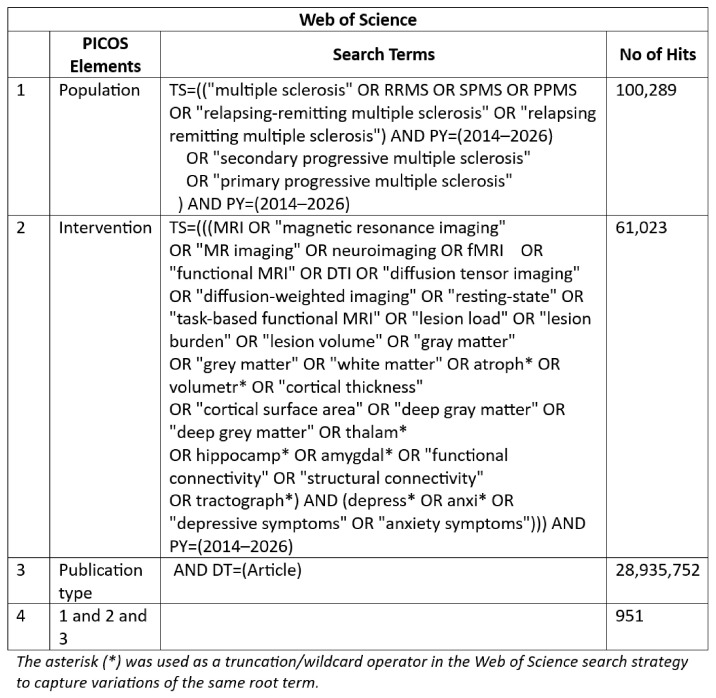
Search strategy and number of hits in Web of Science.

**Figure A4:**
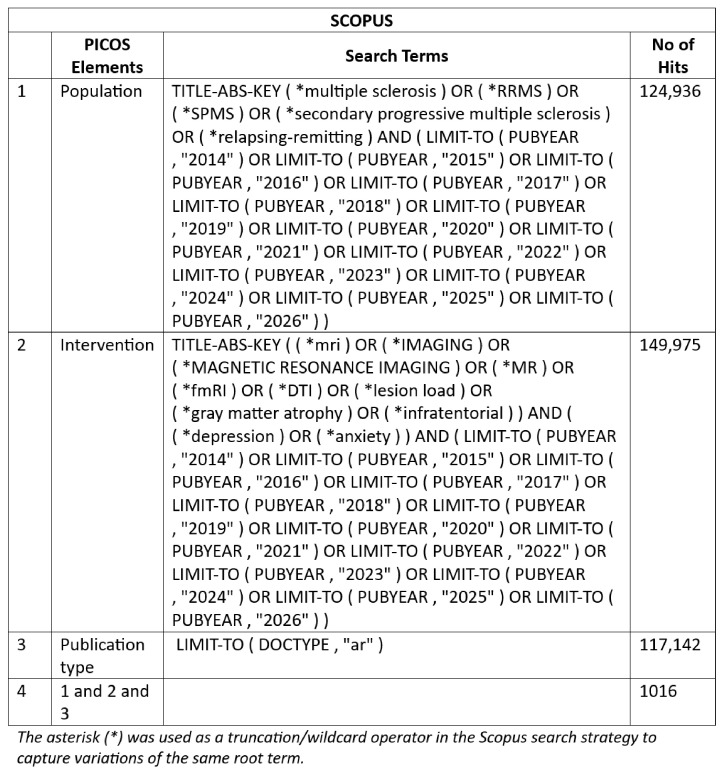
Search strategy and number of hits in Scopus.

## Data Availability

No new data were created or analyzed in this study. Data sharing is not applicable to this article.

## Abbreviations

The following abbreviations are used in this manuscript:

ACC	Anterior Cingulate Cortex
ADC	Apparent Diffusion Coefficient
AFDtot	Apparent Fiber Density Total
AUDIT	Alcohol Use Disorders Identification Test
BDI	Beck Depression Inventory
BDI-II	Beck Depression Inventory-II
BOLD	Blood Oxygen Level-Dependent
CES-D	Center for Epidemiologic Studies Depression Scale
CMDI	Chicago Multiscale Depression Inventory
CSF	Cerebrospinal Fluid
DIR	Double Inversion Recovery
DMN	Default Mode Network
DSM-V	Diagnostic and Statistical Manual of Mental Disorders, Fifth Edition
DTI	Diffusion Tensor Imaging
DWI	Diffusion-Weighted Imaging
ECN	Executive Control Network
EDSS	Expanded Disability Status Scale
EPI	Echo Planar Imaging
FA	Fractional Anisotropy
FC	Functional Connectivity
FLAIR	Fluid-Attenuated Inversion Recovery
fMRI	Functional Magnetic Resonance Imaging
FSS	Fatigue Severity Scale
FWF	Free Water Fraction
Gad+	Gadolinium-Enhancing
GGT	Gamma-Glutamyl Transferase
GM	Gray Matter
GRE	Gradient Recalled Echo
HADS	Hospital Anxiety and Depression Scale
HADS-A	Hospital Anxiety and Depression Scale—Anxiety
HADS-D	Hospital Anxiety and Depression Scale—Depression
HARDI	High Angular Resolution Diffusion Imaging
HDRS	Hamilton Depression Rating Scale
HPA	Hypothalamic–Pituitary–Adrenal
HRQoL	Health-Related Quality of Life
MADRS	Montgomery–Åsberg Depression Rating Scale
MD	Mean Diffusivity
MMSE	Mini-Mental State Examination
MP-RAGE	Magnetization Prepared Rapid Gradient Echo
MPR2RAGE	Magnetization Prepared 2 Rapid Gradient Echoes
MPRAGE	Magnetization Prepared Rapid Acquisition Gradient Echo
MRI	Magnetic Resonance Imaging
MS	Multiple Sclerosis
N/A	Not Applicable
NAWM	Normal-Appearing White Matter
NHPT	Nine-Hole Peg Test
NuFO	Number of Fiber Orientations
OFC	Orbitofrontal Cortex
PCC	Posterior Cingulate Cortex
PD	Proton Density
PFC	Prefrontal Cortex
PHQ-2	Patient Health Questionnaire-2
PHQ-9	Patient Health Questionnaire-9
PROMIS	Patient-Reported Outcomes Measurement Information System
PwMS	People with Multiple Sclerosis
RD	Radial Diffusivity
ROI	Region of Interest
RRMS	Relapsing–Remitting Multiple Sclerosis
rs-fMRI	Resting-State Functional Magnetic Resonance Imaging
S-ANX	State Anxiety
SN	Salience Network
SPMS	Secondary Progressive Multiple Sclerosis
STAI	State-Trait Anxiety Inventory
STAI-T	State-Trait Anxiety Inventory—Trait
STAIS-5	State Anxiety Inventory Short Form
STAIT-5	Trait Anxiety Inventory Short Form
SVR	Support Vector Regression
T-ANX	Trait Anxiety
T1W	T1-Weighted
T2LL	T2 Lesion Load
T2W	T2-Weighted
UF	Uncinate Fasciculus
VBM	Voxel-Based Morphometry
WM	White Matter
WML	White Matter Lesion
WMLL	White Matter Lesion Load

## Appendix A. Research Framework

### Appendix A.1. Research Question and Objectives

The objective of this review was to highlight the link between structural, microstructural and functional brain MRI markers and depression and/or anxiety in patients with MS. The review question was structured according to the PICOS framework (Figure A1). We focused on patients with confirmed MS, independent of disease phenotype or treatment status (Population), who received structured clinical interviews, were assessed using validated psychometric tools, or had established diagnostic criteria. They were linked to measures derived from MRI (Intervention/Exposure). Healthy controls, patients without depression and/or anxiety, lower symptom severity, or within-cohort comparisons based on affective symptom scores were used as comparators. The outcomes of interest were the associations between depression or anxiety and MRI markers including lesion load and topography, cortical thickness, cortical surface area, regional and global brain volumes, gray- and white-matter abnormalities, diffusion metrics, tract integrity, functional activation, and structural or functional connectivity (Outcomes). The eligibility criteria were original adult human observational or interventional studies. The exclusion criteria were non-English articles, reviews, abstracts, editorials, conference papers, letters, errata, case reports, pediatric or non-human studies, studies without MRI-psychiatric correlation analyses, and studies in which patients with MS were only a small subgroup of a heterogeneous cohort (Study design).

### Appendix A.2. Search Strategy

The database-specific search strategies were structured according to the PICOS framework. The detailed search terms, filters, date restrictions, and number of records obtained for each database are summarized in Figure A2, Figure A3 and Figure A4.
